# The gut microbiota is a major regulator of androgen metabolism in intestinal contents

**DOI:** 10.1152/ajpendo.00338.2019

**Published:** 2019-11-05

**Authors:** Hannah Colldén, Andreas Landin, Ville Wallenius, Erik Elebring, Lars Fändriks, Maria E. Nilsson, Henrik Ryberg, Matti Poutanen, Klara Sjögren, Liesbeth Vandenput, Claes Ohlsson

**Affiliations:** ^1^Centre for Bone and Arthritis Research, Department of Internal Medicine and Clinical Nutrition, Institute of Medicine, The Sahlgrenska Academy, University of Gothenburg, Gothenburg, Sweden; ^2^Department of Clinical Chemistry, Sahlgrenska University Hospital, Gothenburg, Sweden; ^3^Department of Gastrosurgical Research and Education, Institute of Clinical Sciences, The Sahlgrenska Academy, University of Gothenburg, Gothenburg, Sweden; ^4^Institute of Biomedicine, Research Centre for Integrative Physiology and Pharmacology and Turku Center for Disease Modeling, University of Turku, Turku, Finland

**Keywords:** dihydrotestosterone, glucuronidation, gut bacteria, intracrinology, testosterone

## Abstract

Androgens exert important effects both in androgen-responsive tissues and in the intestinal tract. To determine the impact of the gut microbiota (GM) on intestinal androgen metabolism, we measured unconjugated (free) and glucuronidated androgen levels in intestinal contents from the small intestine, with a low bacterial density, and from cecum and colon, with a high bacterial density. Using a specific, sensitive gas chromatography-tandem mass spectrometry method, we detected high levels of glucuronidated testosterone (T) and dihydrotestosterone (DHT) in small intestinal content of mice of both sexes, whereas in the distal intestine we observed remarkably high levels of free DHT, exceeding serum levels by >20-fold. Similarly, in young adult men high levels of unconjugated DHT, >70-fold higher than in serum, were detected in feces. In contrast to mice with a normal GM composition, germ-free mice had high levels of glucuronidated T and DHT, but very low free DHT levels, in the distal intestine. These findings demonstrate that the GM is involved in intestinal metabolism and deglucuronidation of DHT and T, resulting in extremely high free levels of the most potent androgen, DHT, in the colonic content of young and healthy mice and men.

## INTRODUCTION

**Fig. 1.**
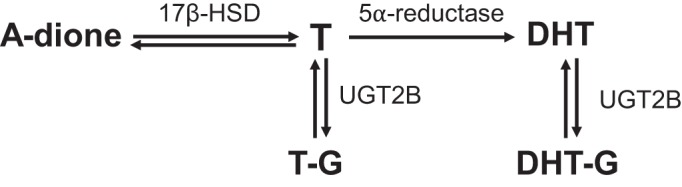
Schematic representation of androgen metabolism. The androgen precursor androstenedione (A-dione) can be reversibly metabolized to testosterone (T) by 17β-hydroxysteroid dehydrogenase enzymes (17β-HSD). T can then be reduced by 5α-reductase enzymes to the more potent androgen dihydrotestosterone (DHT). Both T and DHT can be glucuronidated (-G) in the liver by uridine diphosphate-glucuronosyltransferase (UGT) 2B enzymes to make them more water soluble and facilitate excretion. Glucuronidation is reversible.

Testis-derived testosterone (T) is the most abundant androgen in the systemic circulation in males, whereas the ovaries are the most important source of androgens in females of reproductive age. Androgen production in the gonads is regulated by gonadotropins luteinizing hormone (LH) and follicle-stimulating hormone (FSH), which in turn are regulated by negative feedback of the steroids produced (4, 8). In humans, the adrenal-derived androgen precursor dehydroepiandrosterone (DHEA) also contributes to androgen synthesis (9). Although DHEA is not produced in mice, the mouse adrenal gland may still also contribute to androgen synthesis via production of the androgen precursor androstenedione (A-dione) (12, 28, 34), which can be converted to T by 17β-hydroxysteroid dehydrogenases (Fig. 1) (8). In target tissues and in the liver, T can be further metabolized by a number of different phase I reactions (e.g., reduction) and phase II reactions (e.g., glucuronidation) (Fig. 1) (41). In certain male reproductive tissues, such as prostate and seminal vesicles, T is converted into the more potent androgen dihydrotestosterone (DHT) by 5α-reductase type 2 enzyme (SRD5a2), whereas in the liver mainly 5α-reductase type 1 enzyme (SRD5a1) converts T into DHT (29). Another homologous enzyme, SRD5a3, has been identified (47), but whether it has the capacity to convert T to DHT is still unclear (13, 14). Both T and DHT can be conjugated, mainly in the liver but also in other tissues, by glucuronidation, which increases the water solubility of the compounds. The glucuronidated androgens are excreted in urine or via bile to the small intestine (6, 8).

The gut microbiota (GM) consists of trillions of bacteria, virus, and fungi that have coevolved with its host and has been described as a virtual endocrine organ that can produce and excrete a number of substances into its host’s bloodstream, thereby affecting host physiology (15). The number of bacteria and the metabolic capacity of the GM are higher in the cecum and colon compared with the small intestine (10). The composition of the GM also changes along the gastrointestinal (GI) tract (24). Certain strains of bacteria have been shown in vitro to have the ability to metabolize androgens, for example, converting T into DHT (45). The physiological relevance of the GM in androgen metabolism and in levels of glucuronidated and free androgens in different regions in the intestine is mostly unknown. In a recent study, a link between the GM and androgen concentrations in mice was observed, with female mice having received male intestinal contents showing increased serum T levels. The local androgen levels in intestinal contents were, however, not analyzed in the study (33).

Germ-free (GF) mice have been successfully used to evaluate the role of the GM in many physiological and pathophysiological conditions such as obesity, osteoporosis, and neuroendocrine disorders (32, 43). They are bred in a sterile environment in isolators and are not colonized by bacteria, fungi, or viruses. To the best of our knowledge, androgen levels in intestinal contents and extraintestinal tissues of GF mice have not previously been thoroughly assessed.

We hypothesized that the GM, with low abundance in the small intestine and high abundance in the distal intestine, might modulate androgen metabolism and thereby affect local androgen levels in a site-specific manner along the GI tract. To enable quantification of androgens in intestinal contents, we modified and validated our specific gas chromatography-tandem mass spectrometry (GC-MS/MS) method previously developed for serum sex steroid analyses (37). We observed major GM-dependent differences in levels of glucuronidated and free androgens in the intestinal contents from the small intestine and the more distal regions of the intestine. We also observed GM-dependent changes in androgen action in extraintestinal tissues. Overall, these findings identify the GM as a major regulator of local androgen action in the intestine as well as in other peripheral tissues.

## MATERIALS AND METHODS

### Animals

Wild-type C57BL/6 mice (*n* = 7 per sex) were purchased from Charles River (Wilmington, MA) and housed in the animal facility at the University of Gothenburg with controlled temperature (22°C) and light cycle (12 h light and 12 h dark). The mice were fed a soy-free pellet diet (Teklad Global 16% diet) and had free access to water. Conventionally raised murine pathogen-free (CONV-R) and GF wild-type C57BL/6 mice (*n* = 10 per group and sex) were housed in Taconic facilities (Germantown, NY) and were fed autoclaved NIH-31M diet. Sterility of GF animals was routinely confirmed by aerobic and anaerobic fecal cultures and 16S bacterial RNA screening via PCR of fecal samples. At 8 wk of age, animals were anesthetized and blood was collected from the axillary vein. The animals were subsequently euthanized by cervical dislocation, and tissues and intestinal wall and contents were immediately collected, weighed, and snap frozen in liquid nitrogen. Blood was coagulated at room temperature for at least 30 min and centrifuged for 5 min, and the serum was collected and snap frozen. All animal experiments were conducted in accordance with all relevant legislation, and the experiments conducted in-house were approved by the ethics committee of the University of Gothenburg (ethical approval no 194-2015).

### Clinical Samples

Serum and feces samples were obtained from eight healthy men aged 23–31 yr [body mass index 24.0 ± 0.6 kg/m^2^ (mean ± SE)] participating in a study on lipid metabolism in the intestine. The study was approved by the regional ethical review board in Gothenburg (ethical approval no 807-11), and written informed consent was obtained from all study participants.

### Androgen Analysis

#### Sample preparation.

Frozen tissues, serum, and intestinal contents were thawed on ice. A sample of 2–100 mg (depending on sample type and size) was weighed and placed in a 2-mL screw-top Eppendorf tube with 450 μL of phosphate-buffered saline. The samples were homogenized by shaking with a 5-mm steel bead in a Tissuelyzer II for 5 min. Serum samples were measured volumetrically by pipetting and adjusted to a volume of 450 μL with deionized water.

#### Deglucuronidation.

We have previously developed a GC-MS/MS assay that measures unconjugated androgens (37). To be able to assess glucuronidated forms of these steroids, we compared free levels of each hormone with and without enzymatic deglucuronidation. All intestinal contents were thus divided into two aliquots before homogenization, and one of the aliquots from each sample was deglucuronidated by adding 50 μL of β-glucuronidase (from *Escherichia coli* K12 in 50% glycerol solution; Roche, Basel, Switzerland), followed by brief vortexing and incubation at 37°C with agitation for 1 h. Samples were then frozen at –80°C until steroid extraction and analysis. The difference between total and free levels was calculated at each site and labeled glucuronidated.

#### Androgen extraction and analysis.

Steroids were extracted, derivatized, and measured as described previously with modifications as outlined below (37). Briefly, after addition of isotope-labeled standards, steroids were extracted by liquid-liquid extraction with 1-chlorobutane, followed by solid-phase extraction with Silica SPE columns (Hypersep Si 500 mg; Thermo Scientific, Waltham, MA) that were washed with ethyl acetate-pentane-heptane [10:45:45 (vol:vol:vol)]. For intestinal content and liver samples three washing steps were used instead of the regular two. Next, the analytes were eluted to isooctane and the organic solvent evaporated. Finally, derivatization was performed in two steps: oximation with pentafluorobenzylhydroxylamine hydrochloride followed by esterification with pentafluorobenzoyl chloride. DHT, T, and A-dione were separated on a gas chromatograph and detected simultaneously with electron capture negative chemical ionization by an Agilent 7000 triple quadrupole mass spectrometer (Agilent, Santa Clara, CA) operating in multiple reaction monitoring mode with ammonia as reagent gas. All peaks were automatically integrated with the MassHunter quantitative analysis workstation software from Agilent. The measured concentration was corrected for the amount of input material (wet mass of intestinal contents and tissues or volume of serum). The assay was validated by spiking samples of intestinal content with different concentrations of isotope-labeled DHT, T, and A-dione, and the accuracy (%) was calculated as (measured androgen − baseline androgen)/spiked androgen × 100. The lower limit of quantification (LLOQ) was defined as the lowest concentration with a coefficient of variation (CV) < 20% and accuracy 80–120%. Precision and accuracy were determined as the mean of four samples (Tables 1 and 2). Serum LH and FSH were measured by time-resolved immunofluorometric assays as previously described (25, 48).

**Table 1.** Precision of assay in intestinal contents

	DHT	Testosterone	Androstenedione
QC-low	12.2% (20)	12.2% (40)	1.0% (7.5)
QC-high	4.9% (200)	0.2% (1,000)	3.1% (375)

Validation of the gas chromatography-tandem mass spectrometry method. Precision is calculated as the coefficient of variation (CV) for the 3 androgens in spiked intestinal contents. Values within parentheses are the concentration for the quality control (QC; in pg/g). Calculated as CV of 4 samples. DHT, dihydrotestosterone; QC-low, QC sample with low concentration; QC-high, QC sample with high concentration.

**Table 2.** Accuracy of assay in intestinal contents

	Spiked, pg/g	Baseline, pg/g	Accuracy, %
DHT			
Low	20	0	114
High	200	0	100
Testosterone			
Low	40	0	109
High	100	0	107
Androstenedione			
Low	7.5	0	85
High	37.5	0	95

Values are accuracy of the gas chromatography-tandem mass spectrometry method used, determined by spiking intestinal content samples with isotope-labeled androgens at low and high concentrations. Calculated as (measured androgen–baseline androgen)/spiked androgen × 100, as mean of 4 samples. DHT, dihydrotestosterone.

Values below the LLOQ were set to the LLOQ. To calculate the levels of glucuronidated steroids, the free level (concentration without glucuronidation) was subtracted from the total level (concentration after deglucuronidation). Since the calculation involved two separate sample measurements, differences smaller than the assay’s CV% could not be detected. Therefore, if the mean difference between total and free levels was <15% of the free value, or below the LLOQ, the glucuronidated levels were denoted not detectable. To make intraintestinal and tissue steroid concentrations comparable to those in the serum, 1 g of tissue or intestinal contents was considered equivalent to 1 mL of serum. Androgen levels in the small intestinal content were analyzed from three separate locations (proximal jejunum, midjejunum, and ileum). The androgen levels were very similar at these locations, and therefore the mean for each androgen at all small intestinal locations with at least 8 mg sample size was calculated for each mouse and referred to as the androgen level in the small intestinal content.

### Real-Time Quantitative PCR

RNA was isolated from muscle (quadriceps), seminal vesicle, liver, and intestinal wall from four parts of the small intestine (duodenum, proximal jejunum, midjejunum, and ileum), cecum, and colon with the RNeasy Mini Kit (Qiagen, Hilden, Germany) according to the manufacturer’s instructions. cDNA was synthesized with the High-Capacity cDNA Reverse Transcription Kit (Applied Biosystems, Foster City, CA). Amplifications were performed with the StepOnePlus Real-Time PCR System (Applied Biosystems) and TaqMan probes (Applied Biosystems), labeled with the reporter fluorescent dye FAM (*Srd5a1*: Mm_00614213_m1, *Srd5a2*: Mm00446421_m1, *Srd5a3*: Mm_04243702_m1). As an internal standard, predesigned primers and probe labeled with the reporter fluorescent dye VIC, specific for 18S ribosomal RNA, were included in the reactions (4310893E; Applied Biosystems). Relative expression was calculated by the ΔΔC_T_ method (where C_T_ is threshold cycle) with 18S as reference gene.

### Statistical Analyses

We used Graph Pad Prism 8 (Graph Pad Software, San Diego, CA) and Microsoft Excel 16 (Microsoft Corp., Redmond, WA) for all statistical analyses. Results are presented as means ± SE. For the comparison of androgen levels in mouse intestinal contents we used nonparametric tests since several measurements were below the LLOQ, making the data not normally distributed. Wilcoxon matched-pairs signed-rank test was used for cecum and colon compared with the small intestine of the same animals, and Mann–Whitney *U* test was used for the comparison between two groups (CONV-R vs. GF). To compare gene expression, androgen levels in tissues, and serum and tissue weights between the two mouse groups (CONV-R vs. GF), Student’s two-tailed unpaired *t* test was used. For the comparison of serum and fecal levels of androgens in humans, two-tailed paired Student’s *t* test was used. (Table 3).

**Table 3.** Overview of comparisons made in the experiments

Figure	Strain, Subjects	*n*	Analyses	Comparison	Statistical Test
2, 3	C57BL/6 wild-type mice, CONV-R	7	Androgens	Cecum and colon vs. small intestine	Wilcoxon pairwise signed rank
4	C57BL/6 wild-type mice, CONV-R	10	Androgens	Different tissues	No statistical test, descriptive
5, 6	C57BL/6 wild-type mice, GF and CONV-R	10	Androgens in intestinal contents	GF vs. CONV-R	Mann–Whitney *U* test
7*D*, 8, 9; Table 4	C57BL/6 wild-type mice, GF and CONV-R	8–10	Androgens, gonadotropins, tissue weights	GF vs, CONV-R	Student’s unpaired *t* test, 2 tailed
10	Young adult men	8	Androgens	Serum vs. feces	Student’s paired *t* test, 2 tailed

*n* = no. per group. CONV-R, conventionally raised; GF, germ free.

## RESULTS

### Comparison of Glucuronidated and Free Androgen Patterns in Small Intestine, Distal Intestine, and Extraintestinal Tissues

To assess the levels of different androgens and their glucuronides in different regions within the GI tract, we first optimized and validated our established GC-MS/MS method (37) for measurement of DHT, T, and A-dione in the intestinal contents. The established assay for intestinal contents had a LLOQ of 20, 40, and 7.5 pg/g for DHT, T, and A-dione, respectively. The method presented excellent precision and accuracy for analyses of androgens in intestinal content (Tables 1 and 2). All three examined androgens were present in intestinal contents but with a site-specific pattern in young adult mice.

**Fig. 2.**
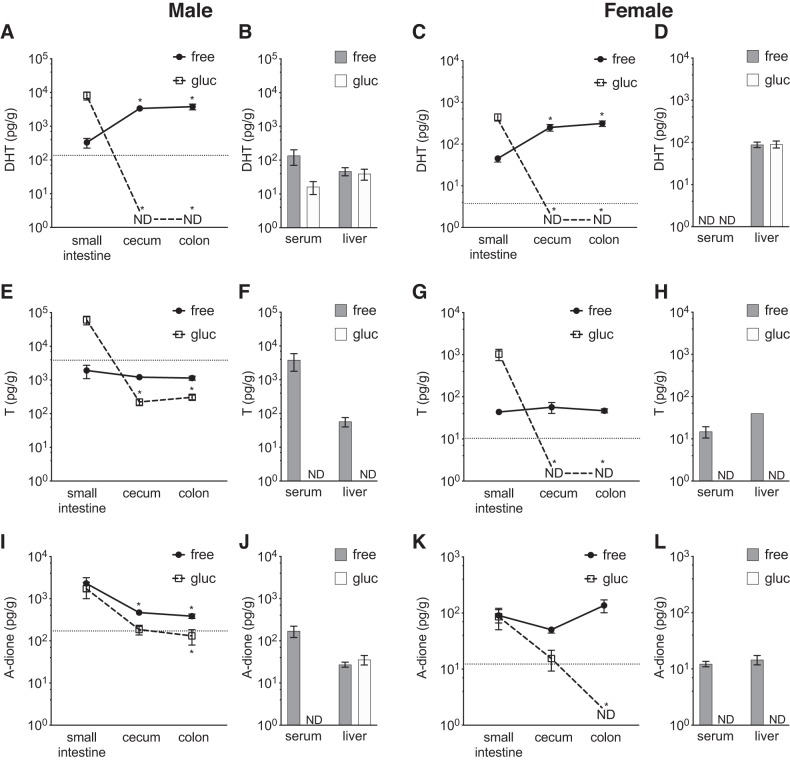
Comparison of glucuronidated and unconjugated androgens in contents of small and distal intestine of mice with normal gut microbiota. Unconjugated (free) and glucuronidated (gluc) androgen levels were measured by gas chromatography-tandem mass spectrometry in different parts of the intestine, serum, and liver of 8-wk-old C57BL/6 mice. *A–D*: dihydrotestosterone (DHT) in males (*A* and *B*) and females (*C* and *D*). *E–H*: testosterone (T) in males (*E* and *F*) and females (*G* and *H*). *I–L*: the androgen precursor androstenedione (A-dione) in males (*I* and *J*) and females (*K* and *L*). Values are shown as means ± SE; *n* = 7 mice. ND, not detectable; dotted horizontal line denotes the corresponding serum free level. In serum the unit of measurement is picograms per milliliter. To compare cecum and colon with small intestine, Wilcoxon matched-pairs signed-rank test was employed. *P < 0.05 vs. small intestine.

**Fig. 3.**
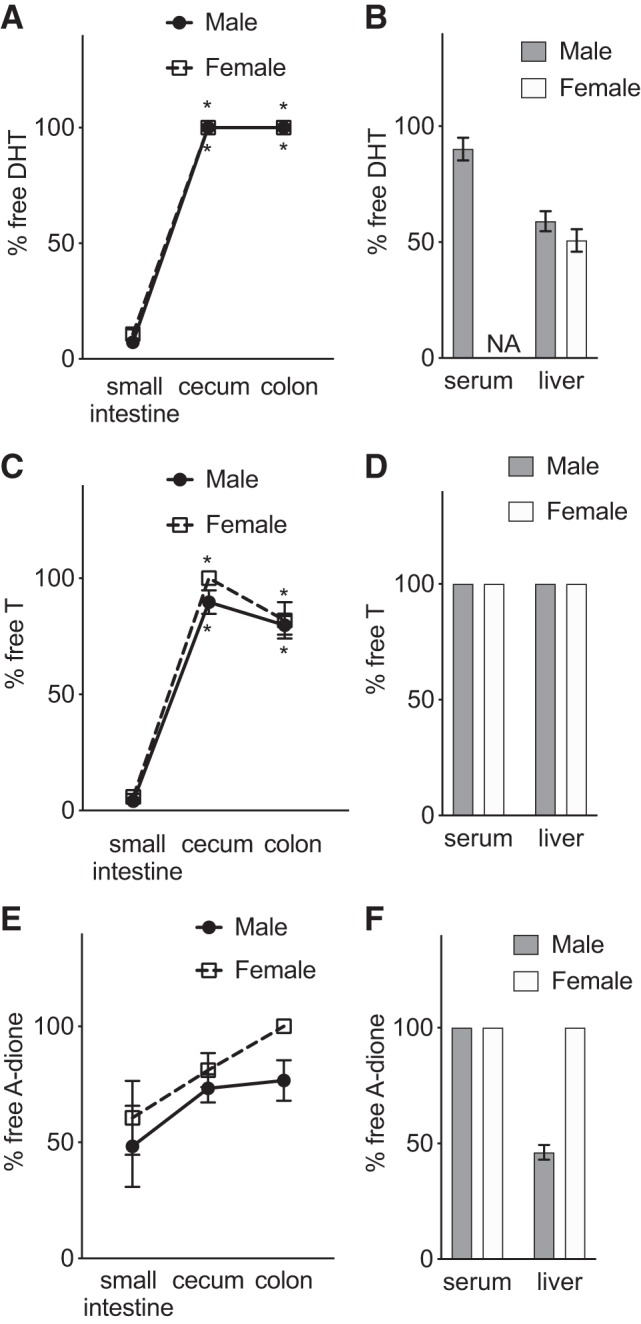
Comparison of unconjugated androgen fraction in small and distal intestine of mice with normal gut microbiota: unconjugated fraction (% free) of dihydrotestosterone (DHT; *A* and *B*), testosterone (T; *C* and *D*), and androstenedione (A-dione; *E* and *F*) in parts of the intestine, serum, and liver of 8-wk-old male and female C57BL/6 mice. Values are shown as means ± SE; *n* = 7 mice. NA, not applicable. To compare cecum and colon with small intestine, Wilcoxon matched-pairs signed-rank test was employed. *P < 0.05 vs. small intestine.

In the small intestine the glucuronidated DHT levels were high compared with colonic and cecal levels, whereas free DHT levels were low in both male and female mice. In contrast, in the cecum and colon glucuronidated DHT levels were undetectable whereas remarkably high levels of free DHT, exceeding the serum levels by >20-fold, were observed in both male and female mice (Fig. 2, *A–D*). The glucuronidated T levels were high in the small intestinal content compared with the very low levels observed in cecal and colonic contents. Free T levels were mainly unchanged between the small intestinal content and the distal intestinal content in cecum and colon in both male and female mice and were similar to the serum levels in male mice and slightly higher than the serum levels in female mice (Fig. 2, *E–H*). In the small intestine nearly all DHT and T were present in the glucuronidated form, whereas in distal regions of the intestine (cecum, colon) nearly all DHT and a majority of T were present in the free form (Fig. 3). The androgen precursor A-dione was present throughout the intestine, with concentrations of the free form higher than in serum in both males and females, whereas it did not show extensive glucuronidation (Fig. 2, *I–L*, and Fig. 3*E*). In general, the intraintestinal patterns of free and glucuronidated T and DHT were similar in females and males, although the females, as expected, had lower levels of androgens at all the regions measured (Fig. 2 and Fig. 3).

**Fig. 4.**
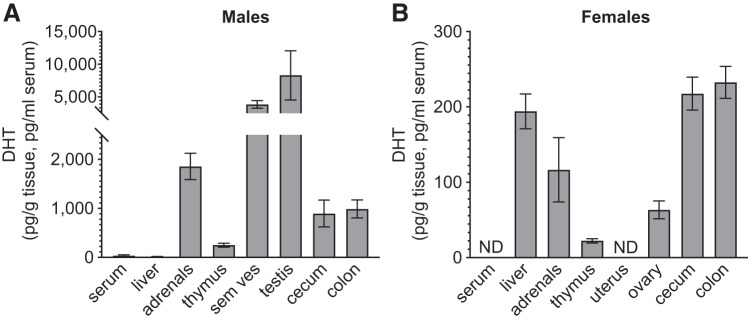
Comparison of unconjugated dihydrotestosterone (DHT) levels in intestine and extraintestinal tissues and serum of mice with normal gut microbiota: DHT levels in intestinal contents, serum, and tissues of 8-wk-old male (*A*) and female (*B*) C57BL/6 mice. Values below lower limit of quantification (LLOQ) are set to LLOQ; if no detectable levels in group, the level is denoted not detectable (ND). Values are shown as means ± SE; *n* = 10 per group. Sem ves, seminal vesicles.

Although male cecal and colonic DHT levels were lower than the levels in testis, they were substantially higher than levels in the liver and thymus and ~25-fold higher than serum DHT levels (Fig. 4*A*). In females, the levels of DHT observed in the cecum and colon were similar to those measured in the liver, whereas clearly lower levels were observed in the ovary and thymus. No detectable DHT levels were observed in the uterus or serum (Fig. 4*B*). Importantly, in female mice free DHT levels in cecum and colon were higher than in most other analyzed tissue (Fig. 4*B*).

### Role of the Gut Microbiota for Glucuronidated and Free Androgens in the Distal Intestine and in Extraintestinal Tissues

**Fig. 5.**
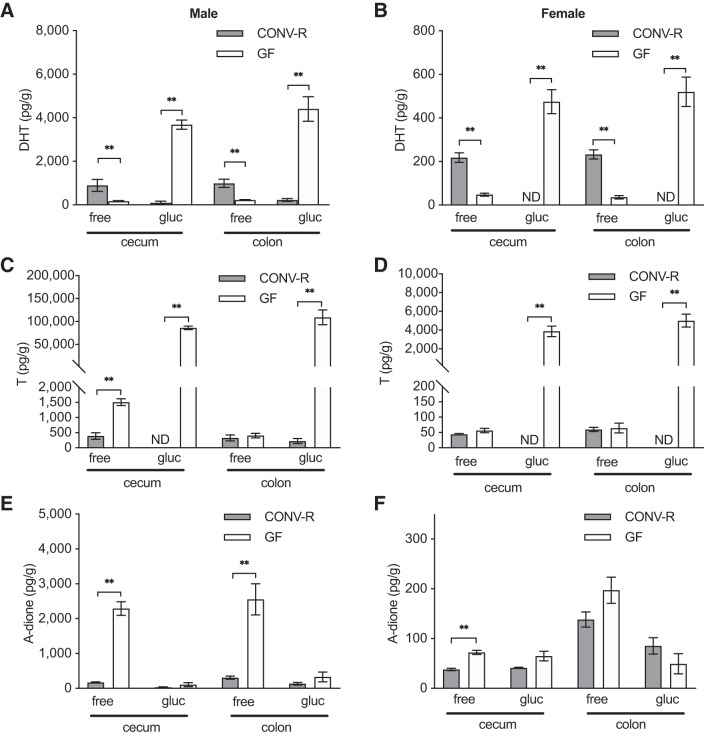
Comparison of glucuronidated and unconjugated androgens in intestinal contents of conventionally raised (CONV-R) and germ-free (GF) mice: unconjugated (free) and glucuronidated (gluc) dihydrotestosterone (DHT; *A* and *B*), testosterone (T; *C* and *D*), and androstenedione (A-dione; *E* and *F*) levels in intestinal contents of 8-wk-old male (*A*, *C*, *E*) and female (*B*, *D*, *F*) CONV-R and GF mice. Values below lower limit of quantification (LLOQ) are set to LLOQ or denoted not detectable (ND). Values are shown as means ± SE; *n* = 10 per group. To compare the 2 groups, the Mann–Whitney *U* test was employed. **P < 0.01.

**Fig. 6.**
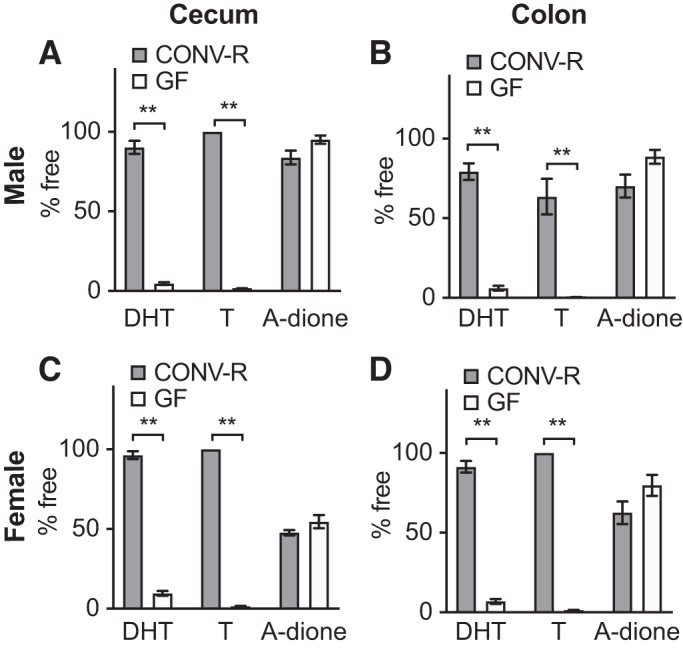
Comparison of unconjugated androgen fractions in the intestine of conventionally raised (CONV-R) and germ-free (GF) mice: unconjugated fraction (% free) of dihydrotestosterone (DHT), testosterone (T) and androstenedione (A-dione) in cecal (*A* and *C*) and colonic (*B* and *D*) contents of 8-wk-old male (*A* and *B*) and female (*C* and *D*) CONV-R and GF C57BL/6 mice. Values are shown as means ± SE; *n* = 10 per group. To compare the 2 groups, the Mann–Whitney *U* test was employed. **P < 0.01.

The findings described above in mice with a normal GM composition suggested that the microbiota present in the distal intestine is responsible for the near-complete deconjugation of DHT and T, causing a substantial difference in glucuronidated and free androgen levels between the small intestine and the cecum. This hypothesis was functionally tested by comparing the levels of glucuronidated and free androgens in GF mice, devoid of gut microbiota, with the corresponding levels in CONV-R mice with a normal GM composition. In both males and females, we observed considerably higher levels of glucuronidated DHT but substantially lower levels of free DHT in both cecal and colonic content of GF mice compared with CONV-R mice (Fig. 5, *A* and *B*). In both sexes, GF mice also had considerably higher levels of glucuronidated T in both cecum and colon than those measured at corresponding sites in CONV-R mice (Fig. 5, *C* and *D*). Unexpectedly, higher levels of both free T and free A-dione were present in cecal content of male GF compared with CONV-R mice (Fig. 5, *C* and *E*) and free A-dione in cecal content of GF female mice (Fig. 5*F*). However, free T levels were not different between GF and CONV-R mice in the colon of male mice or in the cecum or colon of female mice (Fig. 5, *C* and *D*). There were large differences between GF and CONV-R mice regarding the free fraction of T and DHT in cecum and colon for both males and females. In GF mice the free fractions of total DHT and T were <5%, whereas the free fractions in CONV-R mice were between 60% and 100% (Fig. 6). No difference in the free fraction of A-dione was found between the GF and CONV-R mice (Fig. 6). These functional studies using GF mice established that the GM is crucial for the deglucuronidation of DHT and T in the distal intestine.

**Fig. 7.**
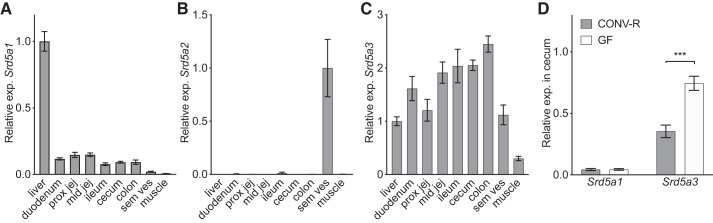
Gene expression of 5α-reductases (*Srd5a1*, S*rd5a2*, *Srd5a3*) in the intestinal tract. *A–C*: gene expression of 5α-reductase type 1 (*A*), type 2 (*B*), and type 3 (*C*) in tissues of 8-wk-old male C57BL/6 mice (*n* = 7). *D*: gene expression of *Srd5a1* and *Srd5a3* in cecum of 8-wk-old germ-free (GF) and conventionally raised (CONV-R) male mice (*n* = 10 per group). Relative expression was calculated with 18S as reference gene. Values are shown as means ± SE. Significant differences between GF and CONV-R according to Student’s 2-tailed unpaired *t* test, ***P < 0.0001. Prox jej, proximal jejunum; mid jej, mid jejunum; sem ves, seminal vesicles.

As levels of free DHT but not free T were increased in cecal content compared with the small intestinal content (Fig. 2, *A–G*), we hypothesized that the GM in cecum might regulate the expression of 5α-reductase enzymes (*Srd5a1*, *Srd5a2*, *Srd5a3*) in the intestinal epithelium. *Srd5a1* was expressed throughout the GI tract at levels around 1/10th of those in the liver used as a positive control (Fig. 7*A*). Also, *Srd5a3* was expressed in the intestinal wall throughout the intestine (Fig. 7*C*), whereas no substantial expression of *Srd5a2* was observed in the intestinal wall in any region of the GI tract compared with the levels observed in seminal vesicles used as a positive control (Fig. 7*B*). The expression of *Srd5a1* was unchanged whereas the expression of *Srd5a3* was increased in the cecal wall of GF mice versus CONV-R mice (Fig. 7*D*). These data did not support the possibility that high 5α-reductase expression could explain the very high levels of free DHT in the distal intestine of CONV-R mice.

**Fig. 8.**
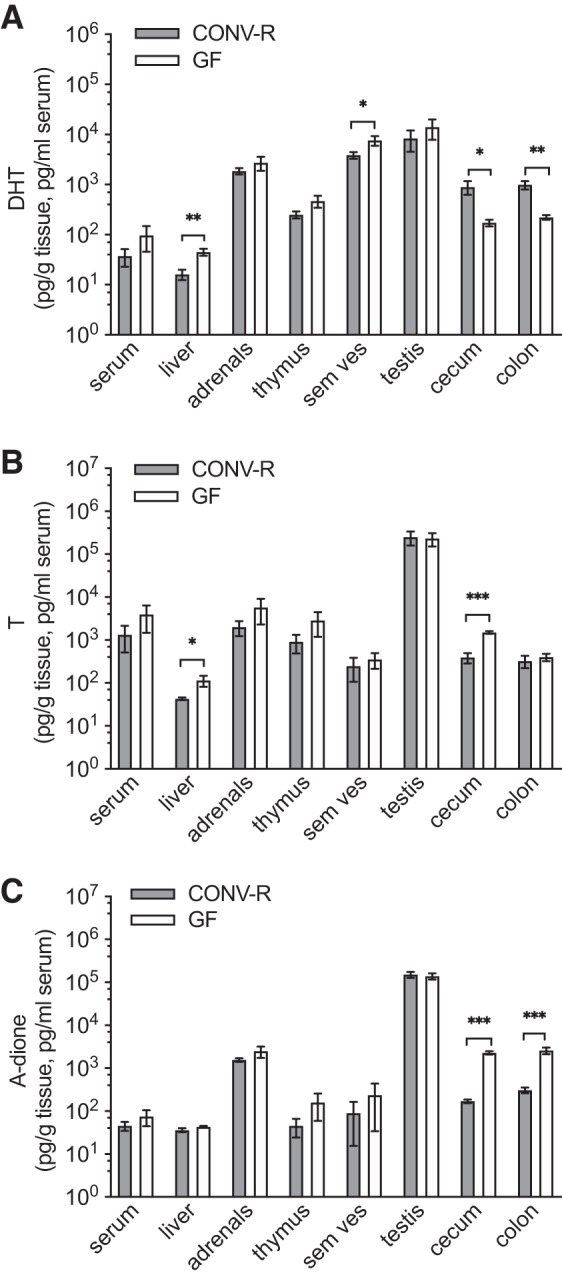
Comparison of unconjugated (free) androgen levels in intestine and extraintestinal tissues of conventionally raised (CONV-R) and germ-free (GF) male mice: dihydrotestosterone (DHT; *A*), testosterone (T; *B*) and androstenedione (A-dione; *C*). Values below lower limit of quantification (LLOQ) are set to LLOQ. Values are shown as means ± SE; *n* = 10 per group. Significance: *P < 0.05; **P < 0.01; ***P < 0.0001 according to Student’s 2-tailed unpaired *t* test. Sem ves, seminal vesicles.

**Fig. 9.**
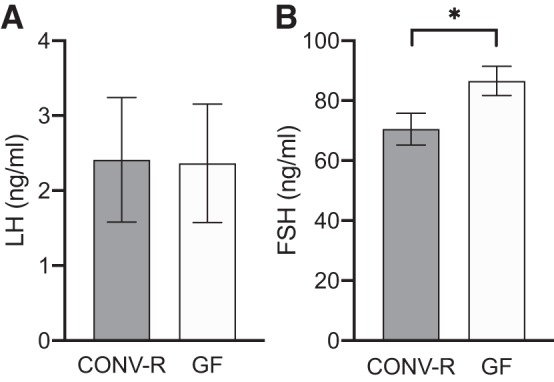
Serum levels of gonadotropins in conventionally raised (CONV-R) and germ-free (GF) male mice: luteinizing hormone (LH; *A*) and follicle-stimulating hormone (FSH; *B*). Values are shown as means ± SE; *n* = 8 per group. Significance: *P < 0.05 according to Student’s 2-tailed unpaired *t* test.

To address the potential effect of GM-regulated androgen metabolism on extraintestinal tissues, we analyzed the androgen content and androgen response in a set of extraintestinal and androgen-responsive tissues in male GF and CONV-R mice. DHT levels in serum did not differ between the groups, but DHT levels in the liver and seminal vesicles were increased in GF compared with CONV-R mice (Fig. 8*A*). T levels in serum did not differ between the groups, and T levels in liver were increased in GF compared with CONV-R mice (Fig. 8*B*), whereas A-dione values did not differ between the groups in serum or the evaluated extraintestinal tissues (Fig. 8*C*). GF males had larger testes than CONV-R males (Table 4). Tissue weights of seminal vesicles were increased in GF mice, whereas the weight of thymus was decreased—both signs of higher androgenic activity in this group (Table 4). To evaluate whether the detected tissue-specific differences in extraintestinal androgen levels and androgen activity in male mice were due to altered feedback regulation of sex steroids, we analyzed LH and FSH in serum from GF and CONV-R male mice. No difference was seen in LH levels, but a significant increase of FSH levels was detected in GF mice compared with CONV-R mice (Fig. 9). Thus glucuronidated DHT levels were substantially higher whereas free DHT levels were lower in the distal intestinal contents of GF compared with CONV-R mice; this was associated with signs of increased androgenic activity in some extraintestinal tissues of male GF compared with CONV-R mice.

**Table 4.** Body and organ weights of conventionally raised and germ-free C57BL/6 mice

	Male		Female	
	Mean ± SE	% Difference	Mean ± SE	% Difference
BW, g				
CONV-R	23.0 ± 0.5	NS	18.8 ± 0.28	NS
GF	23.9 ± 0.5		18.8 ± 0.50	
Liver, % of BW				
CONV-R	4.87 ± 0.14	–26%***	4.81 ± 0.11	–15%**
GF	3.58 ± 0.05		4.10 ± 0.19	
Gonadal fat, % of BW				
CONV-R	0.91 ± 0.06	NS	0.51 ± 0.03	–60%***
GF	0.88 ± 0.05		0.20 ± 0.02	
Thymus, % of BW				
CONV-R	0.19 ± 0.008	–30%***	0.27 ± 0.01	NS
GF	0.13 ± 0.007		0.25 ± 0.01	
Testes, % of BW				
CONV-R	0.72 ± 0.05	+20%*		
GF	0.87 ± 0.03			
Seminal vesicles, % of BW				
CONV-R	0.58 ± 0.03	+14%*		
GF	0.65 ± 0.02			

Values are shown as means ± SE; *n* = 10 mice per group. BW, body weight; CONV-R, conventionally raised; GF, germ free. Significance according to Student’s unpaired 2-tailed *t* test denoted as nonsignificant (NS) = *P* > 0.05,

* *P* < 0.05,

** *P* < 0.01,

*** *P* < 0.0001.

### Comparison of Unconjugated Levels of DHT and T in Feces and Serum of Young Adult Men

**Fig. 10.**
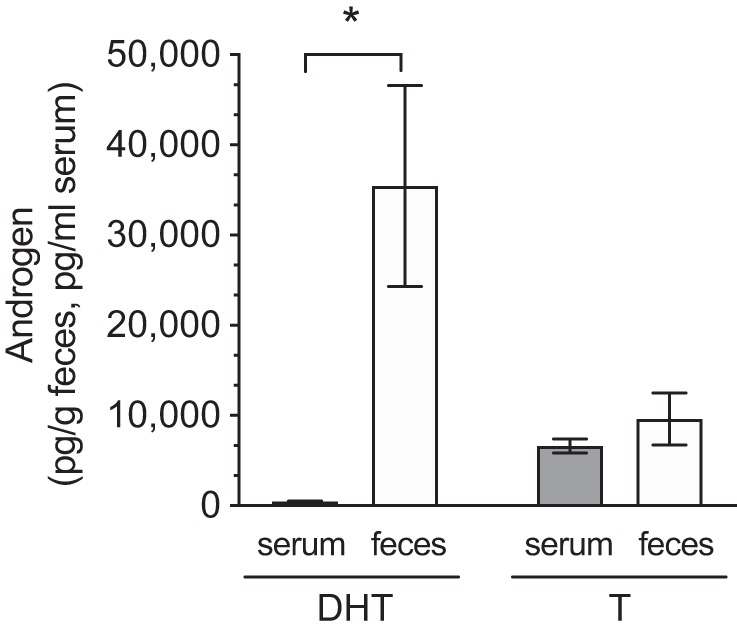
Comparison of unconjugated androgen levels in feces and serum of young adult men. Unconjugated (free) dihydrotestosterone (DHT) and testosterone (T) levels were measured by gas chromatography-tandem mass spectrometry. Values are shown as means ± SE; *n* = 8 subjects. *P = 0.016 according to Student’s 2-tailed paired *t* test.

To translate the main finding of remarkably high unconjugated DHT levels in the distal intestinal content of mice to human physiology, the levels of DHT and T were evaluated in serum and feces from eight healthy young adult men. Similar to our findings in mice, levels of unconjugated DHT, but not T, in feces substantially exceeded the corresponding serum values in these men (DHT: 74-fold higher; Fig. 10). Levels of glucuronidated DHT and T in feces were not detectable with the method used.

## DISCUSSION

Androgens exert important physiological effects, and disrupted androgen action has been associated with the pathophysiology of a number of diseases in both men and women. Although the physiological and pathophysiological roles of the GM in metabolism have been extensively studied during recent years, no previous study has evaluated the role of the GM in androgen metabolism in the intestinal content. Using our optimized and validated GC-MS/MS method to analyze androgen concentrations in intestinal contents, we demonstrated that androgens are to a large extent present in the glucuronidated form in the small intestine. Bacteria in the cecum are crucial for the deglucuronidation of androgens, resulting in remarkably high free DHT levels (compared with serum levels) in the distal intestinal content of both male and female mice. This intriguing finding was also translated to humans, as similarly high free DHT levels were observed in feces of healthy young adult men.

In male and female mice with a normal GM composition we observed that most of the DHT and T was present in the glucuronidated form in the small intestine whereas most of the DHT and T was present in the free form in the distal intestine (cecum and colon). As the number of bacteria and the metabolic activity of the GM in the distal intestine are substantially higher than in the small intestine (24), we hypothesized that bacteria present in cecum are responsible for the deglucuronidation of DHT and T. We therefore investigated the role of the GM in androgen metabolism in the distal intestine with the GF mouse model completely lacking GM. GF mice had very high levels of glucuronidated DHT and T in cecum, in the range of what was found in the small intestine of CONV-R mice. This novel finding demonstrates that the GM in cecum is crucial for deglucuronidation of both DHT and T. It is likely that bacterial β-glucuronidase activity is responsible for this deglucuronidation, as certain members of the GM exhibit high β-glucuronidase activity (19, 20). It has been shown that the capacity of deglucuronidation can vary between individuals (19) and that it can be altered by treatment with probiotics or a change in diet (21, 35). Collectively, these findings demonstrate that the GM in cecum is crucial for the substantial deglucuronidation of DHT and T at this site in young and healthy mice.

The efficient deglucuronidation of DHT in cecum resulted in free DHT levels in cecum and downstream in colon that were considerably higher than serum DHT levels. Remarkably, in female mice the levels of free DHT in the distal intestinal content were higher than in any of the other tissues evaluated, except for the liver. In contrast, GF mice without bacteria in cecum had low levels of free DHT in the distal intestinal content. To exclude the possibility that high levels of 5α-reductase activity in the distal intestinal wall contributed to the observed high free DHT levels in the distal intestinal content of mice with a normal GM, we analyzed *Srd5a1–3* mRNA levels in the intestinal wall. None of these transcripts was highly expressed at this site, arguing against a substantial 5α-reductase activity in the distal intestinal wall. However, we cannot exclude the possibility that local conversion of T into DHT partially contributed to the high free DHT levels in the distal intestinal content. Similar to our findings in mice, levels of unconjugated DHT in feces substantially exceeded the serum DHT values in healthy young adult men, demonstrating that not only mice but also humans have very high unconjugated DHT levels in the distal intestinal content.

In contrast to free DHT, free T levels were not elevated in cecum or colon of mice with normal GM, with free T levels in the distal intestinal content that were similar to serum T levels. Previous studies have shown that intestinal tissue can metabolize T into A-dione, DHT, and other metabolites (16, 17, 44). This metabolic capacity could at least partly explain why the extensive deglucuronidation of glucuronidated T did not result in high free T levels in the cecal content. Furthermore, differences in the transportation of DHT and T via the portal vein or via the intestinal lymphatic system to the systemic circulation might contribute to the observed differences in the ratios between the intestinal content and serum for DHT and T (40). A-dione was present throughout the GI tract, but mainly in its unconjugated form. This finding is most likely explained by the fact that A-dione must be converted to androstanedione before glucuronidation (7).

The finding of very high free DHT levels in the distal intestinal content of mice with a normal GM composition and men was unexpected, and the physiological and pathophysiological roles of DHT within the distal intestine are mainly unknown. DHT has been reported to increase smooth muscle contractility in colon, an action dependent on the androgen receptor (22, 23). Furthermore, androgens can influence intestinal endothelial function (5). It is thus justified to hypothesize that the high levels of DHT, being the most potent androgen, could have a role in the regulation of the contractility and permeability of the distal intestinal wall. Our novel finding of high free DHT levels in the distal intestinal content of mice and men may have implications also for the pathophysiology of intestinal diseases. In humans, the age-adjusted incidence of colon cancer is higher in men than in women (1, 18), and a study in rats has shown that orchidectomy decreases the risk of colonic adenomas and T treatment of orchiectomized rats increases this risk (3). In addition, irritable bowel syndrome (IBS) has been reported to be more common in women than in men (31), and it has been suggested that androgens protect against IBS (27). Furthermore, male patients with IBS show a symptomatology different from that in women, with more men reporting diarrhea whereas constipation is more common in women (11, 36). One may speculate that pharmacological or nutritional treatments affecting the GM composition may change the free DHT levels in the distal intestine and thus have an impact on the risk and pathophysiology of intestinal diseases having a link to androgens, such as colon cancer or IBS.

We also determined the androgen levels and androgenic responses in serum and extraintestinal tissues of GF mice. Using a gold standard MS-based method, we did not observe any significant changes in serum T levels in 8-wk-old male GF C57BL/6 mice compared with CONV-R mice. This is in contrast with previous studies reporting decreased serum T levels in male GF mice compared with mice with a normal GM composition (33, 46, 49, 50). Also, several of these studies indicated that the sexual dimorphism in serum T levels is attenuated in GF mice (33, 49, 50). These studies used less specific immunoassay-based techniques for analysis of serum T levels, with known limitations in the lower concentration range (26, 30), and different mouse strains, most probably with different GM compositions, at different ages compared with the present study. Interestingly, our study demonstrated elevated tissue DHT levels in the liver and seminal vesicles of male GF mice, with a similar trend in the thymus. These data were accompanied by signs of enhanced androgen action in seminal vesicles (increased seminal vesicle weight) and thymus (reduced thymus weight) and an increase in serum FSH. The increased FSH levels in GF mice could contribute to the observed larger testis, as FSH regulates the number of Sertoli cells during development (38). However, previous studies, using GF mice on other genetic backgrounds (outbred strains NMRI and JcI:ICR, respectively) and from other animal facilities with most likely distinct GM compositions in the control mice, did not detect any difference in testis weight between GF mice and control mice (2, 42). FSH has been shown to regulate 5α-reductase activity (39), and this might have contributed to the increased tissue levels of DHT in liver and seminal vesicles in GF mice in the present study. Thus our findings might indicate that the GM has the capacity to modulate androgen metabolism and action also at extraintestinal locations, but further studies are required to clarify the mechanism.

Our study has strengths but naturally also limitations. Our validated mass spectrometry-based detection method for analyses of androgens in intestinal contents is both sensitive and specific, avoiding misclassification of androgens. One limitation with the GF mouse model is that it, for example, has an underdeveloped immune system, and as the mice have been lacking GM from birth we cannot exclude possible confounding developmental effects (32). We did not analyze androgen levels of small intestinal contents from GF mice.

In conclusion, the findings in the present study demonstrate that the GM in the cecum deglucuronidates high levels of glucuronidated DHT and T found in the small intestinal content. This results in remarkably high free levels of the potent androgen DHT in the distal intestinal content of healthy young mice of both sexes and men. We propose that treatments with probiotics or changes in the diet affecting the GM composition might modulate intestinal androgen metabolism and thereby affect the risk of androgen-related diseases mainly in the distal intestine, but potentially also at extraintestinal locations. Further studies are needed to evaluate the physiological and pathophysiological roles of the high levels of free DHT in the distal intestine.

## GRANTS

This study was supported by the Swedish Research Council and the Swedish Foundation for Strategic Research, grants from the Swedish state under the agreement between the Swedish government and the county councils, the ALF agreement (grant numbers 724251, 813251, and 720331), the Lundberg Foundation, the Torsten Söderberg Foundation, the Novo Nordisk Foundation, and the Knut and Alice Wallenberg Foundation.

## DISCLOSURES

No conflicts of interest, financial or otherwise, are declared by the authors.

## AUTHOR CONTRIBUTIONS

H.C., A.L., V.W., L.F., M.E.N., M.P., K.S., L.V., and C.O. conceived and designed research; H.C., V.W., E.E., L.F., M.E.N., K.S., and L.V. performed experiments; H.C., A.L., M.P., K.S., L.V., and C.O. analyzed data; H.C., A.L., V.W., H.R., M.P., L.V., and C.O. interpreted results of experiments; H.C. prepared figures; H.C., L.V., and C.O. drafted manuscript; H.C., V.W., E.E., L.F., H.R., M.P., K.S., L.V., and C.O. edited and revised manuscript; H.C., A.L., V.W., E.E., L.F., M.E.N., H.R., M.P., K.S., L.V., and C.O. approved final version of manuscript.

## References

[1] Abotchie PN, Vernon SW, Du XL Gender differences in colorectal cancer incidence in the United States, 1975-2006. J Womens Health (Larchmt) 21: 393–400, 2012. doi:10.1089/jwh.2011.2992. 22149014 PMC3321677

[2] Al-Asmakh M, Stukenborg JB, Reda A, Anuar F, Strand ML, Hedin L, Pettersson S, Söder O The gut microbiota and developmental programming of the testis in mice. PLoS One 9: e103809, 2014. doi:10.1371/journal.pone.0103809. 25118984 PMC4132106

[3] Amos-Landgraf JM, Heijmans J, Wielenga MC, Dunkin E, Krentz KJ, Clipson L, Ederveen AG, Groothuis PG, Mosselman S, Muncan V, Hommes DW, Shedlovsky A, Dove WF, van den Brink GR Sex disparity in colonic adenomagenesis involves promotion by male hormones, not protection by female hormones. Proc Natl Acad Sci USA 111: 16514–16519, 2014. doi:10.1073/pnas.1323064111. 25368192 PMC4246303

[4] Anderson RC, Newton CL, Anderson RA, Millar RP Gonadotropins and their analogs: current and potential clinical applications. Endocr Rev 39: 911–937, 2018. doi:10.1210/er.2018-00052. 29982442

[5] Ba ZF, Yokoyama Y, Toth B, Rue LW 3rd, Bland KI, Chaudry IH Gender differences in small intestinal endothelial function: inhibitory role of androgens. Am J Physiol Gastrointest Liver Physiol 286: G452–G457, 2004. doi:10.1152/ajpgi.00357.2003. 14563675

[6] Barbier O, Bélanger A Inactivation of androgens by UDP-glucuronosyltransferases in the human prostate. Best Pract Res Clin Endocrinol Metab 22: 259–270, 2008. doi:10.1016/j.beem.2008.01.001. 18471784

[7] Basit A, Amory JK, Prasad B Effect of dose and 5α-reductase inhibition on the circulating testosterone metabolite profile of men administered oral testosterone. Clin Transl Sci 11: 513–522, 2018. doi:10.1111/cts.12569. 29877607 PMC6132360

[8] Bélanger A, Pelletier G, Labrie F, Barbier O, Chouinard S Inactivation of androgens by UDP-glucuronosyltransferase enzymes in humans. Trends Endocrinol Metab 14: 473–479, 2003. doi:10.1016/j.tem.2003.10.005. 14643063

[9] Bélanger B, Bélanger A, Labrie F, Dupont A, Cusan L, Monfette G Comparison of residual C-19 steroids in plasma and prostatic tissue of human, rat and guinea pig after castration: unique importance of extratesticular androgens in men. J Steroid Biochem 32: 695–698, 1989. doi:10.1016/0022-4731(89)90514-1. 2525654

[10] Berg RD The indigenous gastrointestinal microflora. Trends Microbiol 4: 430–435, 1996. doi:10.1016/0966-842X(96)10057-3. 8950812

[11] Björkman I, Jakobsson Ung E, Ringström G, Törnblom H, Simrén M More similarities than differences between men and women with irritable bowel syndrome. Neurogastroenterol Motil 27: 796–804, 2015. doi:10.1111/nmo.12551. 25817301

[12] Callewaert F, Venken K, Kopchick JJ, Torcasio A, van Lenthe GH, Boonen S, Vanderschueren D Sexual dimorphism in cortical bone size and strength but not density is determined by independent and time-specific actions of sex steroids and IGF-1: evidence from pubertal mouse models. J Bone Miner Res 25: 617–626, 2010. doi:10.1359/jbmr.090828. 19888832

[13] Cantagrel V, Lefeber DJ, Ng BG, Guan Z, Silhavy JL, Bielas SL, Lehle L, Hombauer H, Adamowicz M, Swiezewska E, De Brouwer AP, Blümel P, Sykut-Cegielska J, Houliston S, Swistun D, Ali BR, Dobyns WB, Babovic-Vuksanovic D, van Bokhoven H, Wevers RA, Raetz CR, Freeze HH, Morava E, Al-Gazali L, Gleeson JG SRD5A3 is required for converting polyprenol to dolichol and is mutated in a congenital glycosylation disorder. Cell 142: 203–217, 2010. doi:10.1016/j.cell.2010.06.001. 20637498 PMC2940322

[14] Chávez B, Ramos L, García-Becerra R, Vilchis F Hamster SRD5A3 lacks steroid 5α-reductase activity in vitro. Steroids 94: 41–50, 2015. doi:10.1016/j.steroids.2014.11.005. 25498908

[15] Clarke G, Stilling RM, Kennedy PJ, Stanton C, Cryan JF, Dinan TG Minireview: Gut microbiota: the neglected endocrine organ. Mol Endocrinol 28: 1221–1238, 2014. doi:10.1210/me.2014-1108. 24892638 PMC5414803

[16] Eik-Nes KB, Stenstad P, Lofthus R Metabolism in vitro of testosterone (T) to 17 beta-hydroxy-5 alpha-androstane-3-one (DHT) and 5 alpha-androstane-3 alpha,17 beta-diol (3 alpha) by the 800 g supernatant fraction of ileum from rats. J Steroid Biochem 19, 1B: 683–686, 1983. doi:10.1016/0022-4731(83)90235-2. 6887890

[17] Farthing MJ, Vinson GP, Edwards CR, Dawson AM Testosterone metabolism by the rat gastrointestinal tract, in vitro and in vivo. Gut 23: 226–234, 1982. doi:10.1136/gut.23.3.226. 6950919 PMC1419639

[18] Ferlitsch M, Reinhart K, Pramhas S, Wiener C, Gal O, Bannert C, Hassler M, Kozbial K, Dunkler D, Trauner M, Weiss W Sex-specific prevalence of adenomas, advanced adenomas, and colorectal cancer in individuals undergoing screening colonoscopy. JAMA 306: 1352–1358, 2011. doi:10.1001/jama.2011.1362. 21954479

[19] Flores R, Shi J, Gail MH, Gajer P, Ravel J, Goedert JJ Association of fecal microbial diversity and taxonomy with selected enzymatic functions. PLoS One 7: e39745, 2012. doi:10.1371/journal.pone.0039745. 22761886 PMC3386201

[20] Gloux K, Berteau O, El Oumami H, Béguet F, Leclerc M, Doré J A metagenomic β-glucuronidase uncovers a core adaptive function of the human intestinal microbiome. Proc Natl Acad Sci USA 108, Suppl 1: 4539–4546, 2011. doi:10.1073/pnas.1000066107. 20615998 PMC3063586

[21] Goldin BR, Swenson L, Dwyer J, Sexton M, Gorbach SL Effect of diet and Lactobacillus acidophilus supplements on human fecal bacterial enzymes. J Natl Cancer Inst 64: 255–261, 1980. doi:10.1093/jnci/64.2.255. 6766508

[22] González-Montelongo MC, Marín R, Gómez T, Díaz M Androgens are powerful non-genomic inducers of calcium sensitization in visceral smooth muscle. Steroids 75: 533–538, 2010. doi:10.1016/j.steroids.2009.09.012. 19800357

[23] González-Montelongo MC, Marín R, Gómez T, Díaz M Androgens differentially potentiate mouse intestinal smooth muscle by nongenomic activation of polyamine synthesis and Rho kinase activation. Endocrinology 147: 5715–5729, 2006. doi:10.1210/en.2006-0780. 16946014

[24] Gu S, Chen D, Zhang JN, Lv X, Wang K, Duan LP, Nie Y, Wu XL Bacterial community mapping of the mouse gastrointestinal tract. PLoS One 8: e74957, 2013. doi:10.1371/journal.pone.0074957. 24116019 PMC3792069

[25] Haavisto AM, Pettersson K, Bergendahl M, Perheentupa A, Roser JF, Huhtaniemi I A supersensitive immunofluorometric assay for rat luteinizing hormone. Endocrinology 132: 1687–1691, 1993. doi:10.1210/endo.132.4.8462469. 8462469

[26] Handelsman DJ, Jimenez M, Singh GK, Spaliviero J, Desai R, Walters KA Measurement of testosterone by immunoassays and mass spectrometry in mouse serum, testicular, and ovarian extracts. Endocrinology 156: 400–405, 2015. doi:10.1210/en.2014-1664. 25365769

[27] Houghton LA, Jackson NA, Whorwell PJ, Morris J Do male sex hormones protect from irritable bowel syndrome? Am J Gastroenterol 95: 2296–2300, 2000. doi:10.1111/j.1572-0241.2000.02314.x. 11007231

[28] Huhtaniemi R, Oksala R, Knuuttila M, Mehmood A, Aho E, Laajala TD, Nicorici D, Aittokallio T, Laiho A, Elo L, Ohlsson C, Kallio P, Mäkelä S, Mustonen MV, Sipilä P, Poutanen M Adrenals contribute to growth of castration-resistant VCaP prostate cancer xenografts. Am J Pathol 188: 2890–2901, 2018. doi:10.1016/j.ajpath.2018.07.029. 30273606

[29] Kaufman JM, Vermeulen A The decline of androgen levels in elderly men and its clinical and therapeutic implications. Endocr Rev 26: 833–876, 2005. doi:10.1210/er.2004-0013. 15901667

[30] Knuuttila M, Hämäläinen E, Poutanen M Applying mass spectrometric methods to study androgen biosynthesis and metabolism in prostate cancer. J Mol Endocrinol 62: R255–R267, 2019. doi:10.1530/JME-18-0150. 30917337

[31] Lovell RM, Ford AC Effect of gender on prevalence of irritable bowel syndrome in the community: systematic review and meta-analysis. Am J Gastroenterol 107: 991–1000, 2012. doi:10.1038/ajg.2012.131. 22613905

[32] Luczynski P, McVey Neufeld KA, Oriach CS, Clarke G, Dinan TG, Cryan JF Growing up in a bubble: using germ-free animals to assess the influence of the gut microbiota on brain and behavior. Int J Neuropsychopharmacol 19: pyw020, 2016. doi:10.1093/ijnp/pyw020. 26912607 PMC5006193

[33] Markle JG, Frank DN, Mortin-Toth S, Robertson CE, Feazel LM, Rolle-Kampczyk U, von Bergen M, McCoy KD, Macpherson AJ, Danska JS Sex differences in the gut microbiome drive hormone-dependent regulation of autoimmunity. Science 339: 1084–1088, 2013. doi:10.1126/science.1233521. 23328391

[34] Matsumoto C, Inada M, Toda K, Miyaura C Estrogen and androgen play distinct roles in bone turnover in male mice before and after reaching sexual maturity. Bone 38: 220–226, 2006. doi:10.1016/j.bone.2005.08.019. 16213803

[35] McIntosh FM, Maison N, Holtrop G, Young P, Stevens VJ, Ince J, Johnstone AM, Lobley GE, Flint HJ, Louis P Phylogenetic distribution of genes encoding β-glucuronidase activity in human colonic bacteria and the impact of diet on faecal glycosidase activities. Environ Microbiol 14: 1876–1887, 2012. doi:10.1111/j.1462-2920.2012.02711.x. 22364273

[36] Mulak A, Taché Y, Larauche M Sex hormones in the modulation of irritable bowel syndrome. World J Gastroenterol 20: 2433–2448, 2014. doi:10.3748/wjg.v20.i10.2433. 24627581 PMC3949254

[37] Nilsson ME, Vandenput L, Tivesten Å, Norlén AK, Lagerquist MK, Windahl SH, Börjesson AE, Farman HH, Poutanen M, Benrick A, Maliqueo M, Stener-Victorin E, Ryberg H, Ohlsson C Measurement of a comprehensive sex steroid profile in rodent serum by high-sensitive gas chromatography-tandem mass spectrometry. Endocrinology 156: 2492–2502, 2015. doi:10.1210/en.2014-1890. 25856427

[38] Pitetti JL, Calvel P, Zimmermann C, Conne B, Papaioannou MD, Aubry F, Cederroth CR, Urner F, Fumel B, Crausaz M, Docquier M, Herrera PL, Pralong F, Germond M, Guillou F, Jégou B, Nef S An essential role for insulin and IGF1 receptors in regulating sertoli cell proliferation, testis size, and FSH action in mice. Mol Endocrinol 27: 814–827, 2013. doi:10.1210/me.2012-1258. 23518924 PMC5416760

[39] Pratis K, O’Donnell L, Ooi GT, Stanton PG, McLachlan RI, Robertson DM Differential regulation of rat testicular 5alpha-reductase type 1 and 2 isoforms by testosterone and FSH. J Endocrinol 176: 393–403, 2003. doi:10.1677/joe.0.1760393. 12630924

[40] Roberts MS, Magnusson BM, Burczynski FJ, Weiss M Enterohepatic circulation: physiological, pharmacokinetic and clinical implications. Clin Pharmacokinet 41: 751–790, 2002. doi:10.2165/00003088-200241100-00005. 12162761

[41] Schiffer L, Arlt W, Storbeck KH Intracrine androgen biosynthesis, metabolism and action revisited. Mol Cell Endocrinol 465: 4–26, 2018. doi:10.1016/j.mce.2017.08.016. 28865807 PMC6565845

[42] Shimizu K, Muranaka Y, Fujimura R, Ishida H, Tazume S, Shimamura T Normalization of reproductive function in germfree mice following bacterial contamination. Exp Anim 47: 151–158, 1998. doi:10.1538/expanim.47.151. 9816490

[43] Sjögren K, Engdahl C, Henning P, Lerner UH, Tremaroli V, Lagerquist MK, Bäckhed F, Ohlsson C The gut microbiota regulates bone mass in mice. J Bone Miner Res 27: 1357–1367, 2012. doi:10.1002/jbmr.1588. 22407806 PMC3415623

[44] Sohlenius-Sternbeck AK, Orzechowski A Characterization of the rates of testosterone metabolism to various products and of glutathione transferase and sulfotransferase activities in rat intestine and comparison to the corresponding hepatic and renal drug-metabolizing enzymes. Chem Biol Interact 148: 49–56, 2004. doi:10.1016/j.cbi.2004.05.001. 15223356

[45] Soory M Bacterial steroidogenesis by periodontal pathogens and the effect of bacterial enzymes on steroid conversions by human gingival fibroblasts in culture. J Periodontal Res 30: 124–131, 1995. doi:10.1111/j.1600-0765.1995.tb01261.x. 7776153

[46] Tung YT, Chen YJ, Chuang HL, Huang WC, Lo CT, Liao CC, Huang CC Characterization of the serum and liver proteomes in gut-microbiota-lacking mice. Int J Med Sci 14: 257–267, 2017. doi:10.7150/ijms.17792. 28367086 PMC5370288

[47] Uemura M, Tamura K, Chung S, Honma S, Okuyama A, Nakamura Y, Nakagawa H Novel 5 alpha-steroid reductase (SRD5A3, type-3) is overexpressed in hormone-refractory prostate cancer. Cancer Sci 99: 81–86, 2008. 17986282 10.1111/j.1349-7006.2007.00656.x PMC11158902

[48] van Casteren JI, Schoonen WG, Kloosterboer HJ Development of time-resolved immunofluorometric assays for rat follicle-stimulating hormone and luteinizing hormone and application on sera of cycling rats. Biol Reprod 62: 886–894, 2000. doi:10.1095/biolreprod62.4.886. 10727257

[49] Weger BD, Gobet C, Yeung J, Martin E, Jimenez S, Betrisey B, Foata F, Berger B, Balvay A, Foussier A, Charpagne A, Boizet-Bonhoure B, Chou CJ, Naef F, Gachon F The mouse microbiome is required for sex-specific diurnal rhythms of gene expression and metabolism. Cell Metab 29: 362–382.e8, 2019. doi:10.1016/j.cmet.2018.09.023. 30344015 PMC6370974

[50] Yurkovetskiy L, Burrows M, Khan AA, Graham L, Volchkov P, Becker L, Antonopoulos D, Umesaki Y, Chervonsky AV Gender bias in autoimmunity is influenced by microbiota. Immunity 39: 400–412, 2013. doi:10.1016/j.immuni.2013.08.013. 23973225 PMC3822899

